# Smoking-related symptomatology in pregnant smokers during ad libitum smoking and following overnight smoking abstinence

**DOI:** 10.1186/s13104-019-4503-x

**Published:** 2019-08-01

**Authors:** Sharon Allen, Katherine Harrison, Ashley Petersen, Jane Goodson

**Affiliations:** 1 Tobacco Research Programs, Department of Family Medicine & Community Health, University of Minnesota, 717 Delaware Street SE, Minneapolis, MN 55414 USA; 2 Division of Biostatistics, School of Public Health, University of Minnesota, 420 Delaware Street SE, Minneapolis, MN 55455 USA

**Keywords:** Pregnancy, Sex hormones, Tobacco, Smoking-related symptomatology

## Abstract

**Objective:**

Current literature suggests there may be a relationship between sex hormones, which dramatically increase during pregnancy, and nicotine use behaviors. We hypothesized that higher progesterone and progesterone:estradiol ratio (P/E_2_) would be associated with less smoking-related symptomatology (SRS), better mood and fewer cigarettes smoked per day (CPD) during ad libitum smoking and following overnight abstinence in pregnant women. Associations between SRS, mood, smoking behavior and sex hormones were estimated using multiple linear regression with adjustment for CPD and pregnancy trimester.

**Results:**

There were 35 second trimester and 42 third trimester participants. Participants mean age was 26.2 (SD: 4.1), they smoked 11.3 CPD (SD: 4.4) and the mean nicotine dependence score was 4.94 (SD: 1.98). There were no statistically significant associations between progesterone levels, estradiol levels, or the P/E_2_ ratio and SRS or mood measures during ad libitum smoking or following overnight abstinence in this sample of pregnant women. Similarly, there were no associations between sex hormone levels and number of CPD smoked during the ad libitum period. Contrary to our hypothesis, we found no significant associations between sex hormones and SRS, mood or smoking behavior in this sample of pregnant women.

*Trial registration* ClinicalTrials.gov (NCT01811225), December 6, 2012

## Introduction

Smoking in pregnancy is a serious public health problem, which can result in negative effects on the fetus [1–3]. A recent review, identified multiple factors, including socio-demographic, smoking behavior-related, and psychological factors that were associated with smoking cessation during pregnancy [4]. Another factor that is unique to pregnancy and may impact smoking behavior is sex hormones.

Both estradiol and progesterone have well documented effects on brain functioning through neurotransmitters affecting the brain reward systems [5, 6]. Cumulative evidence in preclinical studies [7–10] and more mixed in clinical studies [11–13] suggest that progesterone may attenuate smoking behavior while estradiol may enhance it. Given that in pregnancy, endogenous progesterone levels increase 20-fold and estradiol levels increase by fivefold [14], this could potentially have an effect on smoking behavior.

No one to our knowledge has looked at the role of sex hormones on smoking behavior in pregnant women who smoke. This study’s aim was to examine the associations between progesterone, estradiol, and the progesterone:estradiol ratio (P/E_2_) and smoking-related symptoms (SRS), mood, and smoking behavior during ad libitum smoking and following overnight abstinence in pregnant women. We hypothesized that there would be an inverse association between level of progesterone and the P/E_2_ and SRS (e.g., craving, withdraw, urges, negative affect, perceived stress), mood (depression), and cigarettes smoked (CPD) during ad libitum smoking and following overnight abstinence.

## Main text

### Methods

#### Study design

This cross-sectional study assessed SRS, mood, CPD, and hormone levels in pregnant smokers in their 2nd or 3rd trimester during ad libitum smoking and following overnight abstinence. Ecological momentary assessment (EMA) was used to collect the assessments of SRS, mood, and CPD throughout 7 days of ad libitum smoking. In-clinic assessments were used to collect this data following overnight smoking abstinence. This study was approved by the University of Minnesota Institutional Review Board prior to data collection. All participants provided informed consent and were compensated for their time and participation in the study.

Inclusion criteria included: ≥ 5 CPD for at least the past year, established prenatal care, single gestation uncomplicated pregnancy with no history of two or more miscarriages, and 12–22 or 32–37 weeks gestation. Due to the risk of miscarriage or delay of prenatal care, first trimester women were not included. Exclusion criteria included: using psychotropic medications and/or using nicotine replacement therapy, other cessation aids, or illicit drugs with the exception of marijuana ≤ 2 times per month.

Participants were screened by phone and eligible participants attended a screening visit where final eligibility was determined. Participants then began their testing period. In an attempt to avoid unintentional coercion to continue smoking, participants who completed the testing period between 12 and 22 weeks gestation were not eligible to complete the testing period again at 32–37 weeks gestation.

The study was powered to detect a moderate association (a correlation of 0.3 or greater) between the hormone level and SRS measure. A sample size of 84 women was determined to have 81% power to detect a correlation coefficient of 0.30 for a test of the association between progesterone and self-reported craving with a two-sided 0.05 significance level. A total of 112 participants (equally split between the two trimesters) were to be enrolled to account for an estimated drop-out rate of 25% based on a previous protocol.

#### Ad libitum SRS, mood, and smoking behavior via ecological momentary assessment

On days 1 through 7 during ad libitum smoking, participants tracked cigarettes smoked and completed daily EMA measures using a study-supplied electronic data capture device. The device delivered a scheduled set of SRS and mood measures three times a day. Given that diurnal changes in SRS and mood occur throughout the day, these assessments were delivered in groups of 3 measures during the following blocks of time: 8 a.m.–12 p.m., 12 p.m.–4 p.m. and 4 p.m.–9 p.m. A total of 20 assessments were available throughout the ad libitum data collection. There were no clinic visits nor blood draws during ad libitum smoking.

#### Following overnight abstinence SRS, mood, and hormones

Following the period of ad libitum smoking participants were asked to stop smoking at 6 p.m. and remain abstinent until they completed the in-clinic assessments the following day. Upon arriving at the clinic, all participants completed in-clinic assessments of SRS and mood, provided a serum hormone sample, and had biological confirmation of overnight abstinence by carbon monoxide (CO) levels. A cutoff of CO ≤ 5 ppm was considered abstinent [15].

#### Measures

All participants reported demographics, smoking behavior (e.g., CPD via Timeline Follow Back; [16]), and nicotine dependence via the Fagerstrom Nicotine Dependence Scale (FTND; [17]). The following validated SRS and mood measures were collected: Minnesota Nicotine Withdrawal Scale (MNWS; [18]), Positive and Negative Affect Scale (PANAS; [19]), Perceived Stress Scale (PSS; [20]), Modified Cigarette Evaluation Questionnaire—ad libitum smoking only (m-CEQ; [21]), Brief Questionnaire of Smoking Urges (QSU; [22], and the Center for Epidemiologic Studies Depression Scale (CESD; [23]).

Serum hormone samples were collected following overnight abstinence and sent for analysis at the University of Southern California Endocrine Research Laboratory. Progesterone and estradiol levels were measured by radioimmunoassay with preceding organic solvent extraction and Celite column partition chromatography. The sensitivity of the progesterone assay was 0.010 ng/ml and the inter-assay coefficient of variation (CV) was 12% at 0.230 ng/ml. For the estradiol assay, the sensitivity was 0.002 ng/ml and the inter-assay CVs were 11%, 13%, and 12% at 0.015, 0.036, and 0.101 ng/ml, respectively.

#### Statistical analysis

We used descriptive statistics to summarize the demographics, baseline characteristics, and hormone levels of the participants. We assessed differences in baseline characteristics and hormone levels by trimester (2nd trimester: 12–22 weeks, 3rd trimester 32–37 weeks) using two-sample t-tests and Chi-square tests. Hormone levels were log transformed prior to analysis. SRS, mood, and CPD measured during the ad libitum period were averaged prior to analysis. Multiple measurements were collected during the ad libitum period to obtain a more precise measure of SRS, mood, and smoking during this period. To test for associations between serum progesterone, estradiol, and the P/E_2_ ratio and SRS and mood during ad libitum smoking, multiple linear regression was used with the outcome of the SRS or mood measure and the log of the hormone measurement as the predictor, adjusting for CPD at screening and pregnancy trimester. Analogous models were used to test for an association following overnight abstinence. We tested for an association between serum progesterone, estradiol, and the P/E_2_ ratio and CPD during ad libitum smoking using a multiple linear regression model with the outcome of CPD and the predictor of the log of the hormone measurement with adjustment for pregnancy trimester. No formal correction for multiple testing was made. However, any results with borderline statistical significance will be interpreted cautiously due to the number of outcomes considered. All analyses were performed using R version 3.4.1.

### Results

#### Sample description

There were 106 pregnant women that started the protocol and 90 women who completed the protocol. The final sample included in this analysis consisted of 77 women (n = 35 in 2nd trimester, n = 42 in 3rd trimester). There were 13 participants excluded from the final analysis due to the following reasons: CO ≥ 5 following the overnight abstinence, chose to discontinue participation, or illness.

The women had a mean age of 26.2 (standard deviation [SD]: 4.1) and smoked 11.3 CPD on average (SD: 4.4) at screening. The sample included women who identified as white (n = 41; 53%), African American (n = 18; 23%), other (n = 18; 24%). A majority of the women had completed at least some college (53%). The mean FTND score was 4.94 (SD: 1.98). The only difference by trimester was that participants in their 2nd trimester smoked more CPD 12.7 (SD: 4.9) compared to 10.2 CPD (SD: 3.7) for participants in their 3rd trimester (p = 0.014). The analysis was adjusted for these differences.

#### Hormone levels

Progesterone and estradiol, but not the P/E_2_ ratio, were significantly higher for the participants in their 3rd trimester compared to participants in their 2nd trimester (progesterone: p < 0.001; estradiol: p < 0.001; P/E_2_: p = 0.49). The median progesterone levels were 52.3 ng/ml (1st quartile [Q1]: 38.3, 3rd quartile [Q3]: 58.0) for the 2nd trimester women and 168.3 ng/ml (Q1: 112.3, Q3: 169.1) for the 3rd trimester women. The median estradiol levels were 5.9 ng/ml (Q1: 4.5, Q3: 7.1) for the 2nd trimester women and 18.7 ng/ml (Q1: 14.4, Q3: 24.4) for the 3rd trimester women. The median P/E_2_ ratios were 8.3 (Q1: 6.6, Q3: 10.8) for the 2nd trimester women and 7.7 (Q1: 5.3, Q3: 11.0) for the 3rd trimester women.

#### Hormones and SRS and mood in ad libitum smoking

**Fig. 1:**
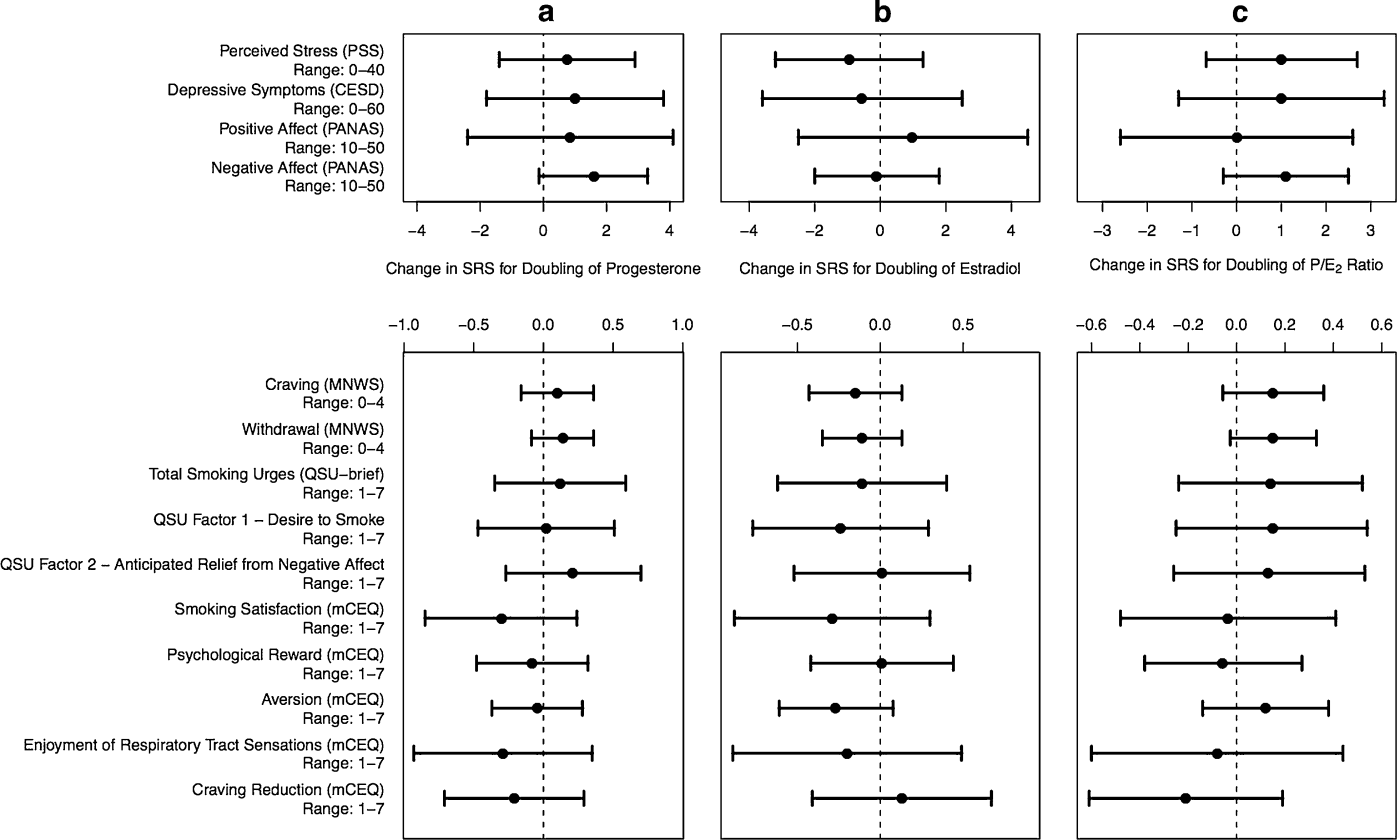
Associations between smoking-related symptomatology (SRS) and mood and levels of **a** progesterone, **b** estradiol, and **c** P/E_2_ ratio during ad libitum smoking, adjusted for cigarettes per day at screening and trimester of pregnancy. The bands indicate 95% confidence intervals. There were no statistically significant associations (p > 0.05 for all measures)

After adjustment for trimester and CPD at screening, there were no statistically significant associations between progesterone levels, estradiol levels, or P/E_2_ ratio and SRS or mood during ad libitum smoking (see Fig. 1).

#### Hormones and SRS and mood following overnight abstinence

**Fig. 2:**
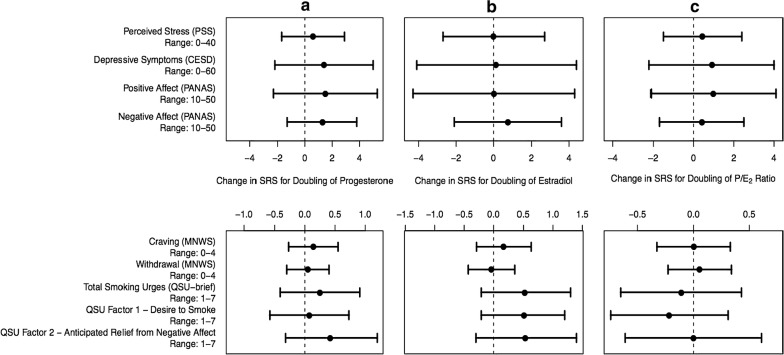
Associations between smoking-related symptomatology (SRS) and mood and levels of **a** progesterone, **b** estradiol, and **c** P/E_2_ ratio after overnight abstinence, adjusted for cigarettes per day at screening and trimester of pregnancy. The bands indicate 95% confidence intervals. There were no statistically significant associations (p > 0.05 for all measures)

After adjustment for pregnancy trimester and CPD at screening, there were no statistically significant associations between progesterone levels, estradiol levels, or P/E_2_ ratio and SRS or mood following overnight abstinence (see Fig. 2).

#### Hormones and cigarettes per day in ad libitum smoking

Comparing those in the same trimester, there were no statistically significant associations between progesterone levels, estradiol levels, or P/E_2_ ratio and CPD during the ad libitum smoking period. Women with twice as high progesterone levels smoked 1.00 more CPD during the ad libitum period (95% CI 0.76 fewer to 2.80 more; p = 0.25). Women with twice as high estradiol levels smoked 0.16 more CPD during the ad libitum period (95% CI 1.90 fewer to 2.20 more; p = 0.87). Women with twice as high P/E_2_ ratio smoked 0.56 more CPD during the ad libitum period (95% CI 0.86 fewer to 2.00 more; p = 0.43).

### Discussion

Our results showed no significant associations between the levels of progesterone, estradiol and P/E_2_ ratio and SRS, mood, and smoking behavior. Based on the literature, we had anticipated that the high levels of progesterone would have some damping effect on SRS and smoking behavior in pregnant women.

The majority of the preclinical work on sex hormones and addictive behavior has been conducted with cocaine and has found that progesterone and its active metabolite allopregnanolone attenuate the rewarding effects of drugs of abuse [24]. Further work has shown that in rats during pregnancy, rising progesterone levels are associated with progressive decline in motivation for cocaine [25] and in nicotine self-administration [26].

Clinical studies are more mixed with regard to the effect of sex hormones on addictive behavior. Mello and colleagues [27] found that progesterone treatment decreased nicotine self-administration under a progressive-ratio schedule suggesting that progesterone decreases nicotine’s reinforcing effects. Reports of SRS symptoms such as tobacco withdrawal are mixed with reports of increased intensity during late luteal phase [28–30] whereas other studies have found no menstrual phase effect on withdrawal [31, 32]. Further studies [12, 33] giving oral exogenous progesterone and IV nicotine showed a trend of decreased smoking behavior, reduced urges to smoke and showed enhanced ratings of “bad effects” from IV nicotine, and attenuated the rating of “drug liking”. One limitation of comparing these studies is the varying methodology related to timing and definition of menstrual cycle phase.

There is sparse clinical data available to evaluate smoking vulnerability at hormonal transition phases such as pregnancy. Evidence suggests that about one half of pregnant women who reported pre-conceptual smoking quit before or during pregnancy [34], but almost 70–80% who achieve abstinence in pregnancy relapse postpartum within 1 year [35]. The role of sex hormones and nicotine addiction in this transition phase when sex hormones are elevated needs further study. Ussher and colleagues [36], compared withdrawal in pregnant and non-pregnant smokers and found that after a 24-h abstinence, the pregnant women had significantly lower mean scores on anger, anxiousness, and impatience after adjusting for baseline cigarette consumption and withdrawal scores. In contrast, a more recent study looked at craving and withdrawal in pregnant and non-pregnant smokers and found that craving scores were higher in pregnant smokers compared to comparison groups and was driven by elevated emotionality and expectancy [37]. It remains unclear why pregnant women are able to quit smoking at a higher rate than non-pregnant women if the pregnancy state does not sufficiently reduce SRS, such as craving and withdrawal.

In summary, this is a first study looking at levels of hormones during pregnancy and their potential association with SRS, mood, and smoking behavior. No significant associations were observed. Further knowledge about the potential role of sex hormones in nicotine addiction is needed to inform cessation strategies.

## Limitations

The limitations of this study included that there was no comparison group, multiple pregnancy factors may override any attenuating benefits of progesterone, and increasing estradiol levels in pregnancy could diminish the attenuating effect of progesterone.

## Data Availability

The dataset used for this project are not publicly available but are available from Dr. Sharon Allen upon reasonable request.

## Abbreviations

P/E_2_: progesterone:estradiol ratio
SRS: smoking-related symptomatology
CPD: cigarettes per day
EMA: ecological momentary assessment
FTND: Fagerstrom Nicotine Dependence Scale
MNWS: Minnesota Nicotine Withdrawal Scale
PANAS: Positive and Negative Affect Scale
PSS: Perceived Stress Scale
m-CEQ: Modified Cigarette Evaluation Questionnaire
QSU: Brief Questionnaire of Smoking Urges
CESD: Center for Epidemiologic Studies Depression Scale
CV: coefficient of variation
CO: carbon monoxide
SD: standard deviation
